# Frequency of off-targeting in genome edited pigs produced via direct injection of the CRISPR/Cas9 system into developing embryos

**DOI:** 10.1186/s12896-019-0517-7

**Published:** 2019-05-06

**Authors:** Kayla Carey, Junghyun Ryu, Kyungjun Uh, Andrea J. Lengi, Sherrie Clark-Deener, Benjamin A. Corl, Kiho Lee

**Affiliations:** 1 Department of Animal and Poultry Sciences, Virginia Tech, Blacksburg, VA 24061 USA; 2 Department of Dairy Science, Virginia Tech, Blacksburg, VA 24061 USA; 3 Large Animal Clinical Sciences, Virginia Tech, Blacksburg, VA 24061 USA

**Keywords:** CRISPR/Cas9, Off-targeting, Genome-editing, Embryo development

## Abstract

**Background:**

The CRISPR/Cas9 system can effectively introduce site-specific modifications to the genome. The efficiency is high enough to induce targeted genome modifications during embryogenesis, thus increasing the efficiency of producing genetically modified animal models and having potential clinical applications as an assisted reproductive technology. Because most of the CRISPR/Cas9 systems introduce site-specific double-stranded breaks (DSBs) to induce site-specific modifications, a major concern is its potential off-targeting activity, which may hinder the application of the technology in clinics. In this study, we investigated off-targeting events in genome edited pigs/fetuses that were generated through direct injection of the CRISPR/Cas9 system into developing embryos; off-targeting activity of four different sgRNAs targeting *RAG2*, *IL2RG*, *SCD5*, and *Ig Heavy chain* were examined.

**Results:**

First, bioinformatics analysis was applied to identify 27 potential off-targeting genes from the sgRNAs. Then, PCR amplification followed by sequencing analysis was used to verify the presence of off-targeting events. Off-targeting events were only identified from the sgRNA used to disrupt *Ig Heavy chain* in pigs; frequency of off-targeting was 80 and 70% on *AR* and *RBFOX1* locus respectively. A potential PAM sequence was present in both of the off-targeting genes adjacent to probable sgRNA binding sites. Mismatches against sgRNA were present only on the 5′ side of *AR*, suggesting that off-targeting activities are systematic events. However, the mismatches on *RBFOX1* were not limited to the 5′ side, indicating unpredictability of the events.

**Conclusions:**

The prevalence of off-targeting is low via direct injection of CRISPR/Cas9 system into developing embryos, but the events cannot be accurately predicted. Off-targeting frequency of each CRISPR/Cas9 system should be deliberately assessed prior to its application in clinics.

**Electronic supplementary material:**

The online version of this article (10.1186/s12896-019-0517-7) contains supplementary material, which is available to authorized users.

## Background

Advancements in genome-editing technology permit us to effectively introduce targeted modifications into non-traditional lab animals, such as pigs. Use of Zinc Finger Nucleases (ZFN) and Transcription Activator-Like Effector Nucleases (TALEN) have shown to be successful [1, 2]; however, they are not as affordable or easy to assemble as the CRISPR (Clustered Regularly Interspaced Short Palindromic Repeats) and Cas9 system [3]. The CRISPR/Cas9 system is used by bacteria in adaptive immunity and defense mechanisms to prevent invasion of foreign genetic material [4]. An engineered CRISPR/Cas9 system, derived from *Streptococcus pyogenes*, could customize the use of the CRISPR/Cas9 system as a powerful genome-editing tool [5].

The CRISPR/Cas9 system has contributed to the production of genetically engineered (GE) pigs by increasing efficiency of genetic modifications in somatic cells and allowing genetic modifications during embryogenesis, thus by-passing the need of somatic cell nuclear transfer (SCNT). Numerous GE pigs have been produced by employing the CRISPR/Cas9 technology [6–8] and have shown a lack of off-targeting activities [9–11]. However, the safety of the approach is still being questioned. Specifically, unintended mutations in the genome caused by off-targeting activity of CRISPR/Cas9 system is a leading concern. Because introducing site-specific double-stranded breaks (DSB) on the target locus is the foundation of many genome-editing tools [12], off-targeting events have been reported with the use of CRISPR/Cas9 system [13–15]. Interestingly, the frequency of off-targeting highly depends on the design of single guide RNA (sgRNA) [3], the complimentary RNA sequence that attracts Cas9 to the target genome locus.

Producing GE pigs through direct injection of CRISPR/Cas9 system into developing embryos provides a unique opportunity. Incorporating SCNT during GE pig production often results in developmental defects. However, GE pigs produced via direct injection of the CRISPR/Cas9 system into developing zygotes appear to be normal and the efficiency is as high as 100% [16, 17]. Pigs are similar to humans in size and physiologically, which makes them a good biomedical model for clinical outcomes [18]. In addition, preimplantation embryos originated from the pig and human display similar development trajectory [19], thus ideal to reflect the outcome of applying CRISPR/Cas9 system in clinics.

Recent reports of genome editing in human embryos [20] and the birth of genome-edited babies [21] suggest that the approach may also be expanded to clinical applications; however, safety of the technology has not been fully assessed. Investigating the presence of off-targeting activities in genome edited pigs produced through direct injection of CRISPR/Cas9 system into developing embryos may reflect the frequency of off-targeting events during clinical applications.

In this study, we investigated off-targeting events in *RAG2/IL2RG* double knockout pigs, *SCD5* knockout fetuses, and *Ig Heavy chain* knockout pigs previously generated via direct injection of the CRISPR/Cas9 system. Frequency of on-target was 100% by the CRISPR/Cas9 systems; however, potential off-targeting activities have not been investigated. The objective of the study was to determine if off-targeting events occurred in knockout pigs by direct injection of CRISPR/Cas9 and examine if the design of sgRNAs affected the occurrence of off-targeting events.

## Results

**Fig. 1:**
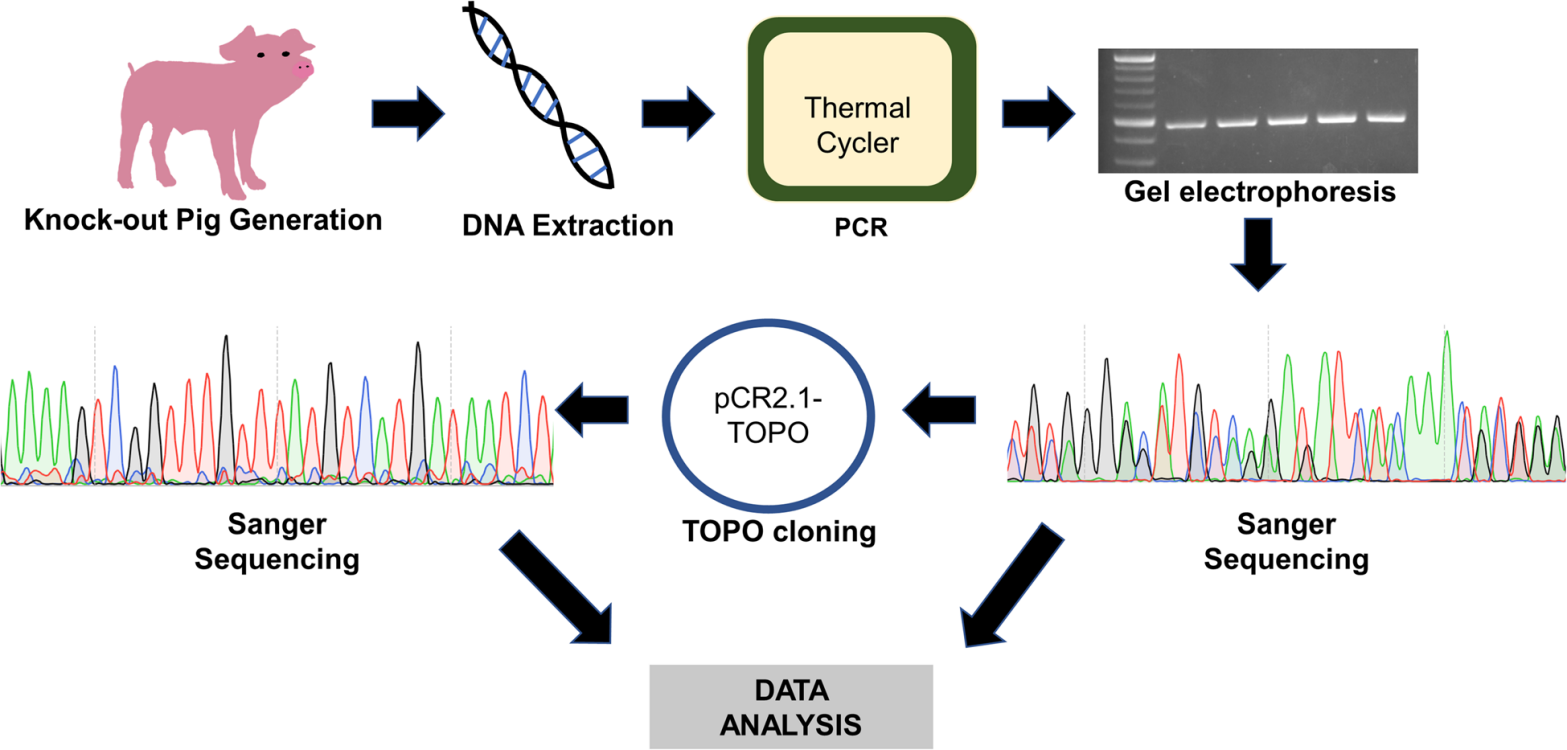
Schematic approaches to identify off-targeting events. First, genomic DNAs were isolated from knockout pigs or fetuses, generated using the CRISPR/Cas9 system. Second, a PCR reaction was performed to amplify a fragment of DNA flanking off-target region. Third, PCR products were loaded on a gel and Sanger sequenced. If the results were polymorphic, then TOPO cloning followed by Sanger sequencing was performed to uncover the genotype of each allele

Bioinformatic analyses identified 9, 8, 6, and 4 potential off-targeting genes carrying 16 bp identity or more with the sgRNA for *RAG2*, *IL2RG*, *Ig Heavy chain,* and *SCD5* knockout pigs respectively. Most of the potential off-targeting loci were located in intron regions and only a portion of the potential off-targeting sites possessed –NGG sequences that can act as a PAM sequence (Additional file 1: Table S1-S4). Presence of off-targeting events on the candidate genes were examined as shown in Fig. 1. Sequencing of potential off-target regions revealed the presence of polymorphisms compared to the *Sus scrofa* reference genome (*Sus scrofa* 10.2 and 11.1). For instance, mismatches were found in sequencing readings from *NTNG1* and 5′ to *LRRF1P1* locus. When compared to the known genomic sequence of *Sus scrofa* breeds and the paternal genomic sequence, the mismatches were identified as polymorphisms due to variations in genomic DNA sequences among breeds. The polymorphism found in the *NTNG1* gene was paternally originated since the same polymorphism exists in the genome of the boar used to generate GE pigs. In addition, a polymorphism in *PLCXD3* was located in a repetitive element of an intron. All the polymorphisms were detected outside of potential sgRNA binding sites. No off-targeting event was detected from sgRNAs used to introduce modifications on *RAG2*, *IL2RG*, or *SCD5* (Additional file 1: Table S5, S6, and S7).

On the contrary, off-targeting events were detected in *AR* and *RBFOX1* genes of *Ig Heavy chain* knockout pigs*.* Mismatches were identified where sgRNA could potentially bind and the presence of a potential PAM site was also located adjacent to the sgRNA binding site. The frequency of off-targeting on *AR* and *RBFOX1* was 80 and 70%, respectively (Table 1). Specific types of off-targeting events are summarized in Table 2. It was found that pigs D and E from *RBFOX1* gene had a biallelic mutation with no wild-type allele. Pig J carried three different genotypes in the *RBFOX1* region with no wild-type sequence present. Genomic DNA of pigs K, L, and M were found to have polymorphisms in the *RBFOX1* gene, while still having the wild-type sequence, indicating that the off-targeting affected only one allele. The *RBFOX1* gene was modified in a homozygous fashion in the genomic DNA of pig C. Pigs A, B, and N showed no off-targeting or polymorphisms in the *RBFOX1* gene. The *AR* gene was altered in biallelic fashion in the genomic DNA of pig E. Pigs B and M carried the wild-type *AR* gene, indicating no off-targeting caused by sgRNA targeting *Ig Heavy chain*. The other pigs examined carried modified *AR* gene in a heterozygous fashion.

**Table 1:** Summary of off-targeting results for *Ig Heavy chain*. Five pigs were tested for each potential off-target gene. Five additional pigs were tested for the *AR* and *RBFOX1* gene. Eight potential off target events were found in the *AR* gene and seven potential off-target events were found in the *RBFOX1* gene. No off-target events were found in the other potential off-target genes

Ig Heavy chain		
Gene	Number of pigs	Number of off-target events (%)
5′ to *LRRF1P1*	5	0 (0%)
*EDIL3*	5	0 (0%)
*AR*	10	8 (80%)
*MYLK3*	5	0 (0%)
*RBFOX1*	10	7 (70%)
*CCDC60*	5	0 (0%)

**Table 2:** Summary of off-targeting events in *AR* gene and *RBFOX1* gene. Ten pigs were analyzed for off-target events. Eight potential off-targets occurred in *AR* gene. Seven potential off-targets occurred in *RBFOX1* gene

*Ig Heavy chain*				
Pig	*AR* gene	WT present?	*RBFOX1* gene	WT present?
WT	TCTCCCCCCAGGTTTTTGTGAGG	N/A	ACACCTCCCAGGCTTTTGTGG	N/A
*A*	TCTCCCCCCAGGTTTTTGTGAGG TCTCCCCCCAGGTTTTTTGTGAGG	yes	ACACCTCCCAGGCTTTTGTGG	yes
*B*	TCTCCCCCCAGGTTTTTGTGAGG	yes	ACACCTCCCAGGCTTTTGTGG	yes
*C*	TCTCCCCCCAGGTTTTTGTGAGG TCTCCCCCCAGGTTTTTTTGTGA	yes	ACACCTCCCAGGCTTGCAGGA	no
*D*	TCTCCCCCCAGGTTTTTGTGAGG TCTCCCCCCAGGTGAGGGAAGA	yes	ACACCTCCCAGGCTTTTGGTG ACACCTCCCAGGCTTGCTGTA	no
*E*	TCT CCCCCCAGGTTTTTGGTGAGG TCTCCCCCCAGGTTTTTGGGGGG	no	ACACCTCCCAGGCTTAGCCTG ACACCTCCCAGGCTGGGGCTT	no
*J*	TCTCCCCCCAGGTTTTTGTGAGG TCTCCCCCCAGGTAATGTAAGTG	yes	ACACCTCCCAGGCTTAGCCTG ACACCTCCCA-----------GCTTGCAGGA ACACCACTAAAATGGGGC	no
*K*	TCTCCCCCCAGGTTTTTGTGAGG TCTCCCCCCAGGTGAGGGAAGA	yes	ACACCTCCCAGGCTTTTGTGG ACACCTCCCACACATCGCCTG	yes
*L*	TCTCCCCCCAGGTTTTTGTGAGG TCTCCCCCCAGGTTTTTTGTGAAG	yes	ACACCTCCCAGGCTTTTGTGG ACACCTCCCAGGCTTCCAGGA	yes
*M*	TCTCCCCCCAGGTTTTTGTGAGG	yes	ACACCTCCCAGGCTTTTGTGG ACACCTCCCAGGCTTGCAGGG	yes
*N*	TCTCCCCCCAGGTTTTTGTGAGG TCTCCCCCCAGGTTTTGGGAAGGG	yes	ACACCTCCCAGGCTTTTGTGG	yes

Following the discovery of off-targeting events in *Ig Heavy chain* knockout pigs, the frequency of off-targeting from a lower concentration of CRISPR/Cas9 system was examined. Genomic DNA isolated from preimplantation embryos injected with 2.5 ng/μL sgRNA and 5 ng/μL Cas9 mRNA (four times lower than the previous concentration) were used to detect off-targeting events on *AR* and *RBFOX1*. On-targeting efficiency to target *Ig Heavy chain* remained at 100%, although one embryo possessed wild-type allele in a heterozygous fashion. Similarly, although similar off-targeting frequency (6/7 for *AR* and 5/7 for *RBFOX1*) was found, there was no biallelic modification on either *AR* or *RBFOX1* and all of the embryos except one (Embryo #3) possessed a wild-type allele (Table 3).

**Table 3:** Summary of genotypes in *Ig Heavy chain* gene, *AR* gene and *RBFOX1* gene in embryos injected with 2.5 ng/μL sgRNA and 5 ng/μL Cas9 mRNA. Seven embryos were analyzed for off-targeting events. Heterozygous: WT and one modified allele; Biallelic: two differently modified alleles; Homozygous: one type of modified alleles; Mosaic: more than two types of alleles and the WT sequence may or may not be present

Embryo #	*Ig Heavy Chain*	*AR*	*RBFOX1*
1	Biallelic	Heterozygous	WT
2	Heterozygous	Heterozygous	Heterozygous
3	Homozygous	Mosaic w/o WT	WT
4	Homozygous	WT	Mosaic w/ WT
5	Biallelic	Heterozygous	Heterozygous
6	Biallelic	Heterozygous	Heterozygous
7	Mosaic w/o WT	Heterozygous	Mosaic w/ WT

Our overall off-targeting analyses were based on 4 sgRNAs used to generate GE pigs/fetuses. However, only one sgRNA resulted in off-targeting events. The low prevalence of sgRNA induced mutations indicates that CRISPR/Cas9 system can be applied to produce GE pigs without causing off-target events.

## Discussion

The CRISPR/Cas9 system utilizes the crRNA, a 20-nucleotide long sequence that is complimentary to the target DNA sequence, to introduce site-specific modifications by inducing DSB on a PAM sequence [13]. Although effective, the CRISPR/Cas9 system can introduce unintended modifications, i.e. off-targeting, that can be permanent if the modifications are introduced into the germ line. Recent application of CRISPR/Cas9 system to correct genetically inherited diseases in human embryos [20] highlights its potential implication as an assisted reproductive technology. However, safety of the approach should be critically analyzed before being applied in clinics and exploring the genome of GE pigs generated through a similar approach (i.e. direct-injection of CRISPR/Cas9 system [16, 17, 22]) can be used to examine potential off-targeting events.

Among twenty-seven genes screened for off-targeting events, only two genes were found to be modified by the CRISPR/Cas9 system. Previous studies indicated that certain mismatches to sgRNAs can lead to inhibition/prevention of off-targeting events [3, 13, 23]; however, our results demonstrate the logic does not consistently apply. For instance, a previous study reported that cleavage activity of SpCas9 was prohibited by only a single-base mismatch up to 11 bp 5′ of the PAM site; however, mismatches further upstream of the PAM did not affect the nuclease activity [3]. On the contrary, although sgRNA targeting *Ig Heavy chain* had a mismatch at position 8 bp 5′ to the PAM of *RBFOX1*, nuclease activity of Cas9 was still active to have caused off-targeting events on the gene. Interestingly, the 5′ to *LRRF1P1* locus also possessed similar mismatches (presence of PAM, mismatches on both off-targeting sensitive and insensitive positions as *RBFOX1*) compared to *Ig Heavy chain* sgRNA; however, there was no nuclease activity. This discrepancy indicates that it may be more challenging to predict off-target events than previously described. In a different study, it was found that mismatches on the 5′ side of the sgRNA of 1–5 base pairs can be tolerated thus causing off-targeting events, whereas mismatches at the 3′ end are less tolerated [13]. Our results were in agreement with this finding. In the case of *AR* gene, there were mismatches on the 5′ end only, and the nuclease activity of Cas9 remained and may have caused potential off-targeting. Another gene, *EDIL3*, also had mismatches only on 5′ end; however no PAM was present, therefore no nuclease activity was observed. These findings suggest that while previous findings are applicable for predicting off-target events, as in the case of *AR* gene, there was random high incidence of off-targeting found in the case of *RBFOX1* gene that did not match previous reports for predicting the likelihood of off-target events.

Although the frequency of off-targeting on *AR* and *RBFOX1* was high (above 70%), the efficiency of on-targeting, i.e. disrupting *Ig Heavy chain* was higher. No wild-type sequence was identified in any of the pigs generated [22], indicating that, although tolerable, mismatches on the 5′ side of sgRNA sequence does reduce the activity of CRISPR/Cas9 system. Similarly, more than two alleles were detected in the *RBFOX1* gene of pig J, suggesting that the off-targeting was introduced after cell division, i.e. first cleavage of embryos. Off-targeting events induced by the CRISPR/Cas9 system are presumably through NHEJ, as the changes in nucleotides were random. Interestingly, most of the embryos that received the low concentration of CRISPR/Cas9 retained at least one wild-type allele (13/14), whereas the frequency (14/20) was lower in embryos that received the higher concentration. Due to the low number of observations, it is difficult to draw a statistically significant conclusion; however, the findings suggest that the prevalence of off-targeting may be decreased with the use of lower concentration of CRISPR/Cas9 RNA. On the other hand, the lower concentration impaired on-target efficiency as one embryo (Embryo #2) carried wild-type sequence of *Ig Heavy chain*, indicating that it is challenging to avoid off-targeting events by lowering concentration of CRISPR/Cas9 system without compromising on-target activity.

Since the oocytes used to generate GE pigs are collected from a local abattoir or purchased from a company, it is impossible to trace genetic background of the maternal side. Therefore, it was difficult to distinguish whether polymorphisms presented through genotyping were due to off-targeting events or naturally occurring polymorphism. Similarly, screening for off-targeting in outbred animals such as humans can be technically challenging because of existing variation in the genome. Traditional inbred animal models such as mice have homozygous genetic backgrounds [24]; however, pigs and humans have diverse genome sequences, which makes it difficult to assess off-targeting events at high accuracy [25, 26]. For instance, conventional surveyor assay and T7E1 analysis [27] may not be able to accurately reflect off-targeting due to the variations in the genome because any allele specific polymorphism may be interpreted as off-targeting events through the assays. Whole genome sequencing can offer a broad map of off-targeting events including unintended locations. However, whole genome sequencing on each genome edited embryo or animal is costly and whole genome sequencing followed by identifying mismatches to the reference genome may not be an absolute solution because any variation from whole genome sequencing needs to be carefully addressed to rule out potential variations from whole genome sequencing [28].

Predicting off-targeting events is technically challenging. Recent developments in computational based web software such as CRISPOR [29] provides a list of potential off-targeting genes that could be affected by designed sgRNA. Interestingly, the website predicts *AR* and *RBFOX1* as potential off-targeting genes linked to the sgRNA used to target *Ig Heavy chain*, demonstrating its effective predictability. However, the website also suggests multiple off-targeting genes related to other sgRNAs targeting *RAG2*, *IL2RG*, or *SCD5*, although no off-targeting events induced by the sgRNAs were identified. This is not surprising as efficiency of CRISPR/Cas9 system can be dramatically affected by the concentrations of CRISPR/Cas9 system delivered into embryos [16]. Therefore, accurately predicting off-targeting events is a challenging process because of variables associated with utilizing CRISPR/Cas9 system to introduce targeted modifications.

## Conclusion

Our study indicates that injecting the CRISPR/Cas9 system into developing embryos results in low incidence of off-targeting events and determining the likelihood of off-target occurrences may be more complex than previous studies have alluded to in outbred animal models. Our findings demonstrate that sequence identities on 5′ side are critical and the presence of a PAM site adjacent to sgRNA is crucial for off-targeting events. However, exceptions to the logic were also identified in our study, indicating that predicting off-targeting can be a challenging process. Off-targeting events reported here were on intron regions, thus unlikely to impact phenotype of resulting GE pigs. However, any unpredicted off-targeting events through clinical application of the technology may result in an irreversible outcome. Unexpected off-targeting events suggest that the technology should be carefully assessed and examined prior to its application.

## Methods

### Bioinformatic analysis to identify potential off-target sites

Potential off-targeting sites were identified as previously described [30, 31]. Specifically, a single-guide RNA (sgRNA) sequence for each gene was compared against the *Sus scrofa* reference genome (10.2 and 11.1) using Basic Local Alignment Search Tool (BLAST) through National Center for Biotechnology Information (NCBI). Annotated genes with at least 16 bp identity with the sgRNA sequence were selected regardless of the presence of possible protospacer adjacent motif (PAM) sequences adjacent to the off-target locations. The summary of potential off-target genes is shown in Additional file 1: Table S1-S4. For the *RAG2*, *IL2RG, SCD5,* and *Ig Heavy chain* knockout pigs, 9, 8, 4, and 6 potential off-target genes were manually identified, respectively. Figure 1 provides an overview of the schematic approach.

### Production of genome-edited pigs

Genomic DNA of GE pigs are in part derived from our previous reports [16, 22] except for *SCD5*. To disrupt *RAG2, IL2RG, Ig Heavy chain*, and *SCD5*, all sgRNAs were designed using a web-based program (http://crispor.tefor.net/) [29]. Specifically, the region of the target gene was submitted through the CRISPOR software and then potential sgRNA were selected among the numerous sgRNAs suggested by the software. Potential target sites by sgRNAs were then BLASTed against the whole pig genome sequence through NCBI to examine specificity of the designed sgRNA. The sgRNAs and Cas9 mRNA were generated through in vitro transcription as previously described [16, 22, 32].

To generate the knockout pigs, first sow ovaries were obtained from a local abattoir or oocytes were purchased from DeSoto. The cumulus oocyte complexes (COCs) were maturated in IVM medium (TCM-199 based media containing FSH and LH, for 42–44 h at 38.5 °C, 5% CO_2_). After maturation, cumulus-free oocytes with a visible polar body were collected for in vitro fertilization (IVF). A group of 28–32 maturated oocytes were transferred into IVF media drops and 50 μl sperm (2.5 × 10^5^ sperm/ml) was introduced into IVF media drops that contained maturated oocytes. The gametes were co-incubated for 5 h at 38.5 °C and 5% CO_2_. After IVF, presumable zygotes were placed in Porcine Zygote Media 3 (PZM-3) and incubated at 38.5 °C, 5% CO_2_, and 5% O_2_ for two hours and then the CRISPR/Cas9 system was injected on the heated stage of a Nikon inverted microscope (Nikon Corporation, Tokyo, Japan). Concentration of CRISPR/Cas9 was 5 ng/uL sgRNA and 20 ng/uL Cas9 mRNA for the generation *RAG2* knockout and 10 ng/uL sgRNA and 20 ng/uL Cas9 mRNA for *Ig Heavy chain, IL2RG,* and *SCD5* knockout generation. Injected zygotes were washed with PZM-3, then cultured in the PZM-3 until embryo transfer (ET). Day 5–6 after IVF, blastocysts and embryos carrying over 16 cells were transferred into surrogate gilts. The embryos were surgically transferred into the oviduct of the gilts. Pregnancy was determined by ultrasound at day 30–35 of gestation. Two embryo transfers were performed to generate 6 *SCD5* fetuses (abortion at embryo day 98) (Additional file 1: Table S8). Genotyping results show that all six fetuses carry modified SCD5 gene without any wild-type sequence (Additional file 1: Table S9).

### Screening for off-targeting events

**Fig. 2:**
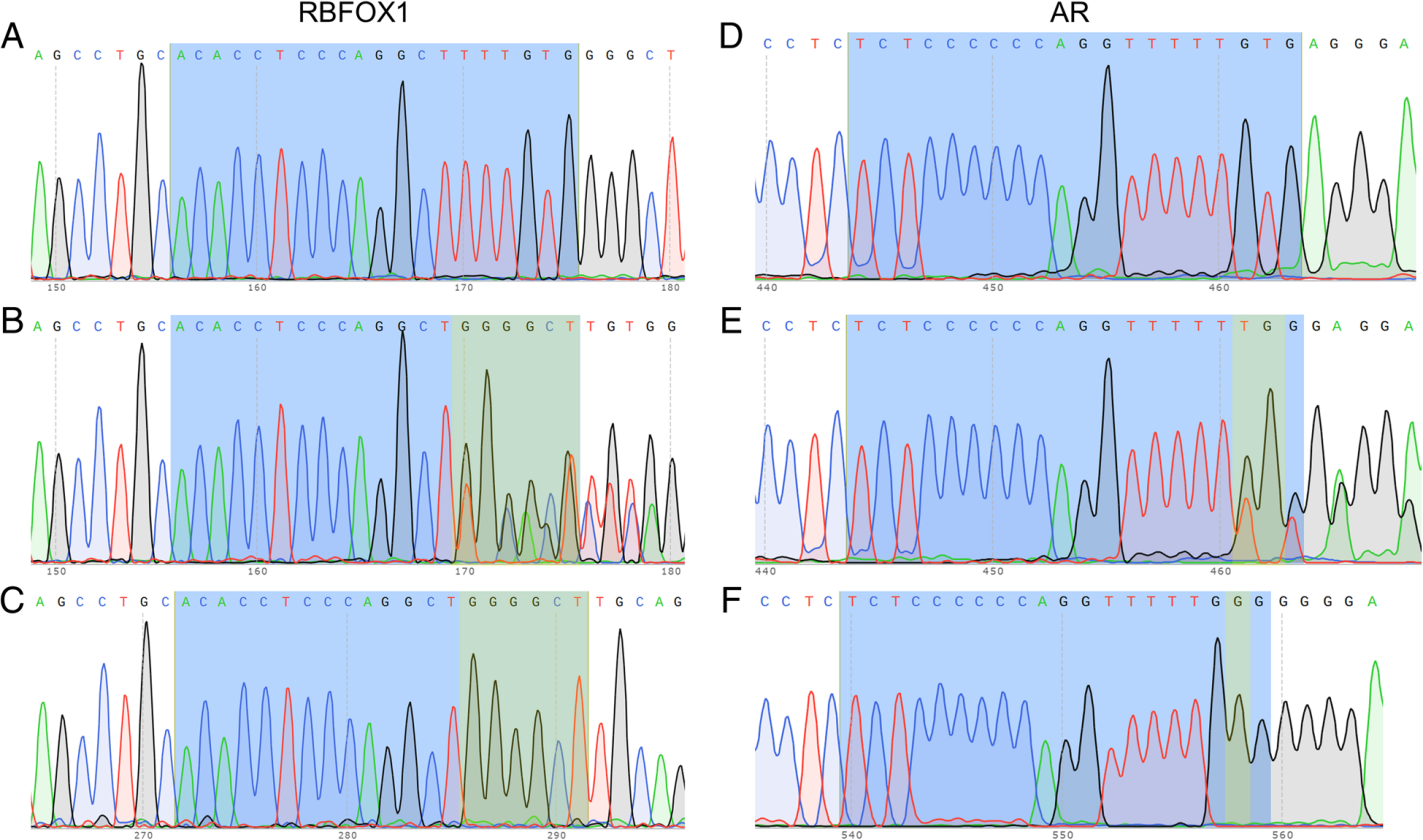
Chromatogram showing potential off-target activity on *RBFOX1* and *AR* gene from *Ig Heavy chain* knockout pig. Similar approach was used to identify any off-target events on *RAG2/IL2RG* double knockout pigs or *SCD5* knockout fetuses. **a** WT sequence of *RBFOX1*. **b** Polymorphic reading from *Ig Heavy chain* knockout pig E. **c** Allele specific reading from *Ig Heavy chain* knockout pig E. **d** WT sequence of *AR*. **e** Polymorphic reading from *Ig Heavy chain* knockout pig E. **f** Allele specific reading from *Ig Heavy chain* knockout pig E. Yellow highlighted portion of sequence indicates deviation from WT sequence reading

Primers were designed to amplify a fragment of genomic DNA flanking potential sgRNA binding sites (off-target sites). The off-targeting events were screened for five *RAG2/IL2RG* double knockout pigs, ten *Ig Heavy chain* knockout pigs, and six 98-day old *SCD5* knockout fetuses. Information on the primers are included in Additional file 1: Tables S10-S13. PCR conditions to amplify off-target regions follow; initial denature at 95 °C for 2 min, denature at 95 °C for 30 s, anneal at 58 °C for 30 s, and extension at 72 °C for 30 s for 34 cycles, final extension at 72 °C for 5 min and holding at 4 °C. Then, PCR amplicons were sequenced at the Biocomplexity Institute of Virginia Tech. Genomic DNA from the boar used for IVF was used as a reference to identify potential sequences of paternally derived alleles. Size of the PCR products ranged from approximately 300 bp to 850 bp. In addition to the PCR sequencing, the PCR products were cloned into a sequencing vector to identify possible off-targeting events on each allele. Specifically, PCR products were cloned into the pCR 2.1 TOPO vector following manufacturer’s instructions. Then, plasmids derived from the cloning were sequenced (Fig. 2).

### Analysis of off-targeting events

Sequencing results identically matching with *Sus scrofa* reference genome were considered to have no off-targeting events. Any mismatches to the reference genome were considered to be potentially due to off-targeting caused by CRISPR/Cas9 system. Sequence readings carrying the mismatches were BLASTed against all known pig genome sequences to investigate whether the mismatches were from known polymorphism sequences, especially in the intron regions.

## Additional file

Additional file 1: **Table S1.** Summary of off-targeting comparison for *RAG2*. Target gene (*RAG2*) guide sequence compared to potential off-target sequence. Location on chromosome, intron or exon region and presence of PAM site is indicated. **Table S2.** Summary of off-targeting comparison for *IL2RG*. Target gene (*IL2RG*) guide sequence compared to potential off-target sequence. Location on chromosome, intron or exon region and presence of PAM site is indicated. **Table S3.** Summary of off-targeting comparison for *Ig heavy chain*. Target gene (*Ig heavy chain*) guide sequence compared to potential off-target sequence. Location on chromosome, intron or exon region and presence of PAM site is indicated. **Table S4.** Summary of off-targeting comparison for *SCD5*. Target gene (*SCD5*) guide sequence compared to potential off-target sequence. Location on chromosome, intron or exon region and presence of PAM site is indicated. **Table S5.** Summary of off-targeting results for *RAG2*. Five pigs were tested for each potential off-target gene. No off-targeting events were found. **Table S6.** Summary of off-targeting results for *IL2RG.* Five pigs were tested for each potential off-target gene. No off-targeting events were found. **Table S7.** Summary of off-targeting results for *SCD5.* Six pigs were tested for each potential off-target gene. No off-targeting events were found. **Table S8.** Embryo transfer to generate SCD5 knockout fetuses. Day 5–6 embryos were transferred into surrogates. A total of 6 fetus were obtained from one surrogate. **Table S9.** Genotype of SCD5 knockout fetuses. No wild-type allele was identified from the fetuses. **Table S10.** Summary of primers used to amplify genes tested for potential off-targeting sites as well as original *RAG2* target gene. **Table S11.** Summary of primers used to amplify genes tested for potential off-targeting sites as well as original *IL2RG* target gene. **Table S12.** Summary of primers used to amplify genes tested for potential off-targeting sites as well as original *Ig heavy chain* target gene. **Table S13.** Summary of primers used to amplify genes tested for potential off-targeting sites as well as original *SCD5* gene. (PDF 451 kb)

## Abbreviations

BLAST: Basic Local Alignment Search Tool
COC: Cumulus Oocyte Complex
CRISPR: Clustered Regularly Interspaced Short Palindromic Repeats
DSB: Double-Stranded Break
ET: Embryo Transfer
GE: Genetically Engineered
IVF: in vitro Fertilization
IVM: in vitro Maturation
NCBI: National Center for Biotechnology Information
PAM: Protospacer Adjacent Motif
PZM-3: Porcine Zygote Media 3
SCNT: Somatic Cell Nuclear Transfer
sgRNA: Single-Guide RNA
TALEN: Transcription Activator-Like Effector Nucleases
WT: Wild-Type
ZFN: Zinc Finger Nucleases
