# Supplementary material for: Frequency of off-targeting in genome edited pigs produced via direct injection of the CRISPR/Cas9 system into developing embryos
Source: BMC Biotechnol. 2019 May 6;19:25. doi: 10.1186/s12896-019-0517-7 (PMC6501304; doi:10.1186/s12896-019-0517-7)
Supplement: Supplementary file 1 — Table S1. Summary of off-targeting comparison for RAG2. Target gene (RAG2) guide sequence compared to potential off-target sequence. Location on chromosome, intron or exon region and presence of PAM site is indicated. Table S2. Summary of off-targeting comparison for IL2RG. Target gene (IL2RG) guide sequence compared to potential off-target sequence. Location on chromosome, intron or exon region and presence of PAM site is indicated. Table S3. Summary of off-targeting comparison for Ig heavy chain. Target gene (Ig heavy chain) guide sequence compared to potential off-target sequence. Location on chromosome, intron or exon region and presence of PAM site is indicated. Table S4. Summary of off-targeting comparison for SCD5. Target gene (SCD5) guide sequence compared to potential off-target sequence. Location on chromosome, intron or exon region and presence of PAM site is indicated. Table S5. Summary of off-targeting results for RAG2. Five pigs were tested for each potential off-target gene. No off-targeting events were found. Table S6. Summary of off-targeting results for IL2RG. Five pigs were tested for each potential off-target gene. No off-targeting events were found. Table S7. Summary of off-targeting results for SCD5. Six pigs were tested for each potential off-target gene. No off-targeting events were found. Table S8. Embryo transfer to generate SCD5 knockout fetuses. Day 5–6 embryos were transferred into surrogates. A total of 6 fetus were obtained from one surrogate. Table S9. Genotype of SCD5 knockout fetuses. No wild-type allele was identified from the fetuses. Table S10. Summary of primers used to amplify genes tested for potential off-targeting sites as well as original RAG2 target gene. Table S11. Summary of primers used to amplify genes tested for potential off-targeting sites as well as original IL2RG target gene. Table S12. Summary of primers used to amplify genes tested for potential off-targeting sites as well as original Ig heavy chain target gene. [file 12896_2019_517_MOESM1_ESM.pdf]

Supplementary Tables

Supplementary Table 1. Summary of off-targeting comparison for *RAG2*. Target gene (*RAG2*) guide sequence compared to potential off-target sequence. Location on chromosome, intron or exon region and presence of PAM site is indicated.

Gene	Sequence	Location on chromosome	Intron/Exon?	PAM
RAG2	GTATAGTCGAGGGAAAAGTA	NC_010444.4 (24547605-24553944)	Exon	Yes
SLCO4C1	GTATAGTTGAGGGAAAATAA	NC_010444.3 (111923785-111924901)	Intron	No
PLCXD3	GTATAGTCAAGGAAAAAGTA	NC_010458.3 (27465590-27466609)	Intron	No
FAM174B	GTATAGTCGAGGGAACCCTC	NC_010449.4 (92221611-92222625)	Intron	No
DPYD	ATATAGTAGAGGGAAAAGTC	NC_010446.4 (132532671-132533688)	Exon	No
DPY19L2	GTATAGTAGAGTGAAAAGTA	NC_010460.3 (42568459-42569440)	Intron	No
UNC13C	ATATAGTGGAGGGAAAAGCC	NC_010443.4 (130045664-130046680)	Intron	No
ITSN2	GTATAGTTGAGGGAAATGTA	NC_010445.3 (121743732-121744717)	Intron	Yes
3' to GNPDA2	ATATAGTCGTGGGAAAAGTT	NC_010450 (35839672-35840689)	Intron	No
3' to LPGAT1	GTATAGGCAAGGGAAAAGTA	NC_010451.4 (131421672-131422691)	Intron	No

Supplementary Table 2. Summary of off-targeting comparison for *IL2RG*. Target gene (*IL2RG*) guide sequence compared to potential off-target sequence. Location on chromosome, intron or exon region and presence of PAM site is indicated.

Gene	Sequence	Location on chromosome	Intron/Exon?	Pam
IL2RG	GGGACTCTCAACGTTTCCAC	NC_010461.5 (57143568-57151242)	Exon	Yes
CCNB3	TGAACTCTGAACGTTTCCAC	NC_010461.4 (48865012-48865996)	Intron	No
ZNF189	TGAACTCTCAACTTTTCCAC	NC_010443.4 (271662850- 271663866)	Intron	No
MYOM1	GGGACTCTCAAAGTTTTAGC	NC_010448.3 (96567814-96568829)	Intron	Yes
P2RX7	GGGACTCTCAATGTTTCTCC	NC_010456.4 (33314885-33315869)	Intron	Yes
NTNG1	GGGACTCTGAACGTTCCCAC	NC_010446.4 (123215716- 123216697)	Intron	No
TRPV1	AGGACTCTCAAGGTTTCCCA	NC_010454.3 (51553951-51554967)	Intron	Yes
5' to LOC102157658	GGGACTCTCTACGTTTCCTG	NC_010461.5 (117490221-117491238)	Intron	No
5' to DCBLD2	GGGACTCTCAATGTTTCCAC	NC_010455.5 (160064437-160156205)	Intron	No

Supplementary Table 3. Summary of off-targeting comparison for *Ig heavy chain*. Target gene (*Ig heavy chain*) guide sequence compared to potential off-target sequence. Location on chromosome, intron or exon region and presence of PAM site is indicated.

**Gene**	**Sequence**	**Location on Chromosome**	**Intron/Exon?**	**Pam**
Ig Heavy Chain	ACACCCCCCAGGTTTTTGTG	NW_018084979.1 (3676148-3841291)	Exon	Yes
5' to LRRF1P1	CAACCCCCCAGCTTTTTGTG	NC_010457 (137255672-137256655)	Intron	Yes
EDIL3	TAACCCCCTAGGTTTTTGTG	NC_010444 (92541026 – 92542043)	Intron	No
AR	TCTCCCCCCAGGTTTTTGTG	NC_010461.5 (53713243- 53714259)	Intron	Yes
MYLK3	ACACCCACCAGGTTTTTGTG	NC_010448.4 (37860344- 37861325)	Intron	No
RBFOX1	ACACCTCCCAGGCTTTTGTG	NC_010445 (34998525-34999544)	Intron	Yes
CCDC60	GCACCCCCTAGGTTTTTGTG	NC_010456 (33512327-33513309)	Intron	No

Supplementary Table 4. Summary of off-targeting comparison for *SCD5*. Target gene (*SCD5*) guide sequence compared to potential off-target sequence. Location on chromosome, intron or exon region and presence of PAM site is indicated.

Gene	**Sequence**	**Location on Chromosome**	**Intron/Exon?**	**PAM**
SCD5	ACCGCTCTGGGTGTGACAGC	NC_010450.4 (135507346-135634031)	Exon	Yes
SDK1	CCTGCTCTGGGTGTGACAGT	NC_010445.3 (3521346-3522331)	Intron	No
KLHL29	AGAGCTCTGGGTGTGGCAGC	NC_010445.3 (122252390- 122253406)	Intron	No
ACLY	CCAGCTCTGGGAGTGACAGC	NC_010454.3 (21092639-21093655)	Intron	Yes
5' to RAB3A	CCCGCTCTGCGTGTGACAGT	NC_010444.4 (59602517-59603534)	Intron	Yes

Supplementary Table 5. Summary of off-targeting results for *RAG2*. Five pigs were tested for each potential off-target gene. No off-targeting events were found.

RAG2		
**Gene**	**Number pigs**	**Number off-target events**
SLCO4C1	5	0
PLCXD3	5	0
FAM174B	5	0
DPYD	5	0
DPY19L2	5	0
UNC13C	5	0
ITSN2	5	0
3' to GNPDA2	5	0
3' to LPGAT1	5	0

Supplementary Table 6. Summary of off-targeting results for *IL2RG*. Five pigs were tested for each potential off-target gene. No off-targeting events were found.

IL2RG		
**Gene**	**Number pigs**	**Number off-target events**
CCNB3	5	0
ZNF189	5	0
MYOM1	5	0
P2RX7	5	0
NTNG1	5	0
TRPV1	5	0
5' to LOC102157658	5	0
5' to DCBLD2	5	0

Supplementary Table 7. Summary of off-targeting results for *SCD5*. Six pigs were tested for each potential off-target gene. No off-targeting events were found.

**SCD5**		
**Gene**	**Number pigs**	**Number off-target events**
SDK1	6	0
KLHL29	6	0
ACLY	6	0
5’ to RAB3A	6	0

Supplementary Table 8. Embryo transfer to generate SCD5 knockout fetuses. Day 5-6 embryos were transferred into surrogates. A total of 6 fetus were obtained from one surrogate.

Surrogate ID	Number of embryos generated	Number of embryos transferred into a recipient	pregnancy	Number of fetuses
27-5	448	65 (day-6)	Yes	6
27-2		50 (day-5)	No	-

Supplementary Table 9. Genotype of SCD5 knockout fetuses. No wild-type allele was identified from the fetuses.

Fetuses	Genotype
1	9 base pair deletion/ 14 base pair deletion
2	2 base pair change, 1 base pair deletion/ 9 base pair deletion
3	14 base pair deletion/ 3 base pair deletion, 1 base pair change
4	4 base pair deletion/ 16 base pair deletion, 3base pair change
5	2 base pair insertion
6	9 base pair deletion, 1 base pair change/ 9 base pair deletion

Supplementary Table 10. Summary of primers used to amplify genes tested for potential off-targeting sites as well as original *RAG2* target gene.

**Gene**	**Forward**	**Reverse**	**Expected Size**
RAG2	AAGGATTCCTGCTACCTTCCTCCT	AGATAGCCCATCTTGAAGTTCTGG	426
SLCO4C1	GTAAACTGATAGGCATTCTAATCCAGAACTAACC	CTGGCCCATCTCCTCCATTC	506
PLCXD3	GAGGGCCGAATCAGTACTTGC	CTCCACAAGACAGGGATGACATC	478
FAM174B	CAGAGGTTGCAGGACAATGGC	GCAATGAACATGCGGGTGC	369
DPYD	CCCTGTGCTTAAAACTCCCACATC	CTAAAGTGACACCGGTTAAGCCC	502
DPY19L2	GGA GTATCTGAAC TCCACTTTTG GGT	AGA AGT GCA ACC AAG GCC AG	496
UNC13C	GTGGGTTTTGTGGGAGTGTAGTG	CCACTGTACTCAAAGAATCCAGATTGAC	410
ITSN2	CAATGAAAGACAGGAAAGACCAGAGGCTG	GCTACAGCATGATGTTCCTTGAGAG	767
3' to GNPDA2	CCTGCTCTGTTCGCCTCAG	CTTTGTACAGAGGTTGGCTCTTCT	575
3' to LPGAT1	GGGGTCTCTTGGTTTGCCTCC	GCTTGTAGGCCGGCTTCTTCAG	463

Supplementary Table 11. Summary of primers used to amplify genes tested for potential off-targeting sites as well as original *IL2RG* target gene.

Gene	Forward	Reverse	Expected Size
IL2RG	CTGGACTATTAGAAGGATGTGGGC	ATATAGTGGGAAGCCTGGGATGCT	417
CCNB3	GCCAGATCCACAGGACACTTTG	GAGGTGACAGCTAAAGGTACAGGG	469
ZNF189	GGCTTTTCTTTCTTCTAGTCTCCACTGG	CCTGGCTGTGAAGTGAACAAATAGTAGG	439
MYOM1	GGTTCCATTCATCTGTCTTTGCTCTC	GGCTGTGGTATAGGCTGGC	452
P2RX7	CTTTGTCACTCTTGCCATCGGG	CTACCCAACAAACTATCCTGAACCTGG	441
NTNG1	GCTGTAATTGGTAAGGCAATGGCTT	GGCCTCTCCCTGCTTCTC	447
TRPV1	GCAGCCTCTGGGTGCTAAG	CCAGGGTGCTTCGCTTGTC	467
5' to LOC102157658	GTGTGCAGATTTCTTCCTATAACGGC	GTTTCAACATTAACATCATGGGGTCCC	400
5' to DCBLD2	CCAGGGTCCCCCTTCCTG	CATGGGTACTGGAACACAAAAGAGGG	466

Supplementary Table 12. Summary of primers used to amplify genes tested for potential off-targeting sites as well as original *Ig heavy chain* target gene.

**Gene**	**Forward**	**Reverse**	**Expected size**
Ig heavy chain	GACACTTTGGAGGTCAGGAACGGGAG	CTTCTCTCTCCGACATGGCTCTTTCAGAC	848
5' to LRRF1P1	GAAGTCACACTCCGCCGGG	GTGAAGAATCTGGAAGGCCCAAGG	390
EDIL3	CATCTTCCTATTCTCCAGCAGGGC	CACATATCACACCTAGAATCTGTGGGAG	462
AR	CTAGAAGATGGTGAATCCTAGTTCCCATTCTC	GAGTGAAGAGGACACTGATATGCCC	650
MYLK3	CACACTCTTCTGTGGTTACTAATGAGAGCC	CCCTGAGACGCCAGGTGG	392
RBFOX1	GCTCTCTCCTCCCTGAGAGG	CAAAGCCATGCTGCCATCAGGAG	401
CCDC60	GCTGTCCTTCTCTCAGTCCCAC	CCTGTGACCTTGGGCAGG	400

Supplementary Table 13. Summary of primers used to amplify genes tested for potential off-targeting sites as well as original *SCD5* gene.

Gene	Forward	Reverse	Expected Size
SCD5	GCAGGAAACAGTCGCAAAGA	GGGGTTACTGTCAGGGTCAA	756
SDK1	GCACGTTTTGTAATGGGGCCG	CCCCCAGTATTTAGCACAGAGCC	352
KLHL29	GGCGCAGCTGGTGTCATAC	GATGCTCACTCTCGAGGGCG	342
ACLY	GGCCAGGGAAAGCAGAGC	CCAGAATTCTAGACGGGGTGGGAG	384
5' to RAB3A	CGAGAGGAGGGCCGTGACAATC	GGACACTCGGGAGCTCACC	467
